# Construction of Progress Prediction Model of Urinary Incontinence in Elderly Women: Protocol for a Multi-Center, Prospective Cohort Study

**DOI:** 10.3390/ijerph19020734

**Published:** 2022-01-10

**Authors:** Di Zhang, Lei Gao, Yuanyuan Jia, Shiyan Wang, Haibo Wang, Xiuli Sun, Jianliu Wang

**Affiliations:** 1 Department of Obstetrics and Gynecology, Peking University People’s Hospital, No. 11, Xi-Zhi-Men South Street, Xi Cheng District, Beijing 100044, China; 1911110388@bjmu.edu.cn (D.Z.); 1911110389@bjmu.edu.cn (L.G.); jiayuan@bjmu.edu.cn (Y.J.); 0062043910@bjmu.edu.cn (S.W.); wangjianliu@pkuph.edu.cn (J.W.); 2 The Key Laboratory of Female Pelvic Floor Disorders, Beijing 100044, China; 3 Research Center of Female Pelvic Floor Disorders, Peking University, Beijing 100044, China; 4 Clinical Research Institute, Peking University, Beijing 100191, China; wanghb_pucri@bjmu.edu.cn

**Keywords:** elderly women with UI, disease progress, prediction model

## Abstract

Background: Urinary incontinence (UI) is a common health problem and seriously affects quality of life. Many women lack understanding of UI or are too ashamed to seek medical advice early, leading to a low treatment rate. The aim of this study is to establish an effective UI progress prediction model for elderly women with UI for earlier detection and better treatment. Methods: This study is conducted as a prospective, multi-center, cohort study, and recruits 800 women aged ≥60 with mild or moderate UI in China. Participants are divided into three groups: stress urinary incontinence group (SUI), urgency urinary incontinence group (UUI), and mixed urinary incontinence group (MUI). This study will investigate the general conditions of patients, after complete relevant pelvic floor function assessment, as well as after follow up at 6 months, 12 months, and 18 months by telephone. The primary endpoint is UI disease progress. Single factor and multi-factor Cox regression model analyses are undertaken to evaluate the associated risk factors affecting the progress of UI to establish a progress prediction model for elderly women. Discussion: This study will provide more predictive information for elderly women with UI, and new clinical references for the intervention and the treatment of UI for medical staff.

## 1. Background

Urinary incontinence (UI) is defined as an abnormal urinary control condition in which urine flows out of the urethral opening involuntarily [1]. It is common lower urinary tract symptom in women and one of the most frequently reported diseases associated with pelvic organ prolapse in adult women. UI can occur in all age groups, whereas it is more commonly seen in middle-aged and elderly patients. The globally reported UI prevalence in women ranges from 25% to 45% and increases year by year [2], and the prevalence of UI among adult women over 60 years old has been reported to be up to 38% [3]. Similarly, the reported UI prevalence in Chinese adult women differs from 8.7% to 69.8%, while that in older Chinese women reaches 16.9% to 61.6% [4]. UI is etiologically and pathophysiologically categorized for three types, as follows: stress urinary incontinence (SUI), urgency urinary incontinence (UUI), and mixed urinary incontinence (MUI) [5]. SUI is the most common type, accounting for 50% of all UI cases [6,7], and is defined as involuntary urinary leakage when physical exertion from coughing, sneezing, or laughing causes pressure to the bladder. Inadequate support of the urethra by the pelvic floor muscles and deficiency of the urethral intrinsic sphincter are the recognized essential mechanisms for UI. The reported UI-associated factors include patient age, body mass index (BMI), genetic factors, smoking, race, constipation, pregnancy and multiple childbirths, previous pelvic floor operations (e.g., hysterectomy), and hormone deficiency or menopause, all of which cause pelvic floor muscle weakness [3,8,9]. UUI prevalence has been reported in many studies to be between 1% to 7% [6,7], which is much lower than UI. UUI generally refers to an overactive bladder caused involuntary urinary leakage with urgency, which is usually caused by involuntary contractions of the bladder wall detrusor muscle. UUI may be triggered by simple daily phenomena and activities such as the sound of tap water, exposure to low temperatures, or drinking cold drinks, and has several congenital causes including myogenic, neurogenic, and urethral symptoms [3,8,10]. MUI is a mixture of symptoms of SUI and UUI, with a reported incidence of 7.5% to 25%, secondary to SUI [6,7]. Its etiology is also a combination of factors causing SUI and UUI [11,12].

UI in women is a huge global health problem. Besides affecting women’s quality of life by limiting social activities, productivity, and social and sexual behaviors, it also has significant impacts on medical costs and even increases financial burden on women and society [4,13]. In addition, regarding the psychological impact of UI on women, numerous research works claim that it creates a long-term period of mental suffering, such as depression or anxiety [14,15,16]. However, some scholars have found, through study of female athletes, that UI does not cause depression and anxiety. Such conclusions are most possibly related to the study subjects, namely female athletes, who have an acknowledged relationship between UI and sport, with most suffering mild UI, as well as having a stronger sense of happiness because of their sport, thus UI might not impact their psychological mode [17].

UI as a common public health issue, is a worldwide problem that has not been indicated to most women, and thus not enough attention has been paid to its early diagnosis and treatment. The impacts of mild and moderate to severe UI on quality of life are greatly different. Although mild UI has little impact on quality of life, as it becomes more severe, it will not only worsen quality of life, but will also bring heavy financial burden to the family and society. Therefore, it is important to use early interventions to upgrade patients’ quality of life and reduce the financial burden related to this disease by detecting any risk factors from patients with mild UI, so as to prevent their mild UI from progressing to moderate or severe.

The common interventions for the prevention of UI include lifestyle intervention, pelvic floor muscle training, biofeedback therapy, and electrical stimulation therapy, etc. However, all these interventions lack standard and systematic evaluations of their preventive effectiveness [18,19].

With the increasing popularity of big data technology, using machine learning to measure the situation and to predict the development of diseases closely related to human health has globally become a hot research topic and application field recently. At present, there is a precedent of using clinical diagnostic software based on large-scale epidemiological data analysis to predict the incidence of diseases in the world. A well-known prognostic model is the Framingham risk score, which predicts the risk of cardiovascular disease in 10 years [20]. Current studies on UI are all disease pathogenesis models, and there are no reports on UI development or the application of any prediction model or software for UI progress in elderly women.

The aims of this study are to systematically investigate the disease progress related factors for elderly women with UI, so as to establish a UI progress prediction model and to improve science-based interventions and preventive measures for UIs [19,21].

## 2. Materials and Methods

### 2.1. Study Design and Setting

**Figure 1:**
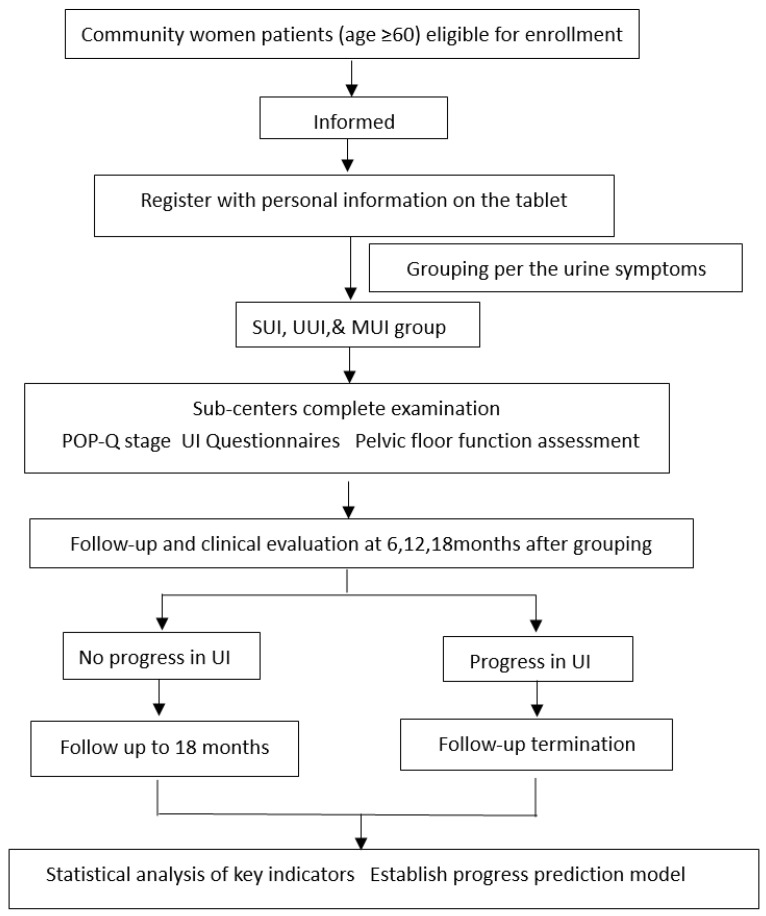
Study flow chart.

The study is conducted as a prospective multicenter study to collect epidemiological factors for the analysis of the impacts of physical activity, chronic diseases, pregnancy and delivery, genetic characteristics, pelvic floor function assessment (e.g., pelvic floor muscle strength, pelvic floor ultrasound, pelvic floor electrophysiology, and morphology index), and other factors in elderly women. Data analysis will be conducted by means of bioinformatics and mathematical modeling objectives to investigate the influence of UI progress and prognosis related factors, and to construct a prediction model of UI progress in elderly women. This study is carried out by Peking University People’s Hospital in Beijing, in cooperation with Wuhan University People’s Hospital in Wuhan, China. A total of 800 patients will be recruited, 400 from Beijing and 400 from Wuhan. Four district-level hospitals in Beijing are involved as the sub-centers in Beijing, namely Tongzhou District Maternal and Child Health Care Hospital, Fengtai District Maternal and Child Health Care Hospital, Fangshan District Maternal and Child Health Care Hospital, and Changping District Maternal and Child Health Care Hospital. Two district-level hospitals in Wuhan are involved as the sub-centers in Wuhan, namely Wuhan Qingshan District Maternal and Child Health Hospital and Wuhan East Lake New Technology Development Zone Fozu Mountain Community Health Service Center. Each sub-center will select communities within the district that are targeted to recruit elderly female patients who are ≥60 years of age with mild to moderate UI by distributing a research subject leaflet. Eligible women need to sign informed consent before being enrolled in the cohorts. Figure 1 illustrates the flow diagram of the study for all subjects.

### 2.2. Participants

The project is approached by phone calls to women ≥60 years of age (the elderly women) and were recently diagnosed with mild or moderate UI. A primary screening survey is conducted during the approach by asking the women three simple questions by investigators: whether they have urine leakage, what is the urine leakage frequency, and what accompanying symptoms they have. Women who are primarily screened to have potential mild and moderate UI will be invited for further examination at the hospitals, where the preliminary examination will be conducted by trained doctors while recording the detailed patient’s history.

#### 2.2.1. Inclusion Criteria

Participants will be enrolled if they are: (1) ≥60 years of age; (2) educated enough to complete the questionnaires; (3) able to move freely to attend a hospital examination; (4) previously diagnosed as mild and moderate UI, including SUI, UUI, and MUI; (5) suffering urine leakage during occasional coughing, exertion, sneezing, fast walking, and other abdominal pressure; or (6) suffering frequent and urgent urination with urinary leakage and nocturia.

#### 2.2.2. Exclusion Criteria

Patients will be excluded from the study if they were diagnosed as having: (1) severe pelvic organ prolapse, (2) serious cardiovascular and cerebrovascular diseases or other life-threatening diseases, (3) malignant tumor threatening patients life, (4) urogenital tract fistula, (5) previous history of anti-UI surgery and medication for UI, (6) no self-care ability and unable to complete the follow-up, and (7) severe UI and chronic urinary retention.

### 2.3. Randomization and Blinding

In this study, patients will be recruited using convenient sampling without randomization. All investigators, other than those conducting recruitments, will be masked in our study.

### 2.4. Informed Consent

All patients will sign the consent forms (two copies) after being fully informed about this study in detail and any questions they had were answered. One copy will be handed over to the patients, while another copy will be stored at the sub-center for unified management.

### 2.5. Intervention

This is an observational prospective cohort study with no intervention to the enrolled patients.

### 2.6. Initial Screening, Assessment, and Follow-Up

After informed consent, participants will be arranged in the hospital in batches. The patients will be received by a team that consists of physicians specialized in the pelvic floor and nurses. All investigators of the team are comparatively fixed and need to be trained by a principal investigator from Peking University People’s Hospital on the research protocol, the inclusive and exclusive criteria, and the questionnaire interviewing. Roleplay on questionnaire interviewing was conducted to have each of the team members interview a provider’s female family member who had no idea about the study, under the supervision of the principal investigator, to confirm that the question was raised using publicly understandable terms and to avoid inducive wording. Questionnaires to the participants will be completed via interview in a doctor’s office at the clinic, which will be specially resettled for allowing participants to be interviewed under a comfortable circumstance so as to avoid answer bias from patient nervousness.

Every participant will be given a unique study ID. Participants’ demographic information, including age, marital status, education level, occupation, BMI, living habits, menstruation, marriage, childbirths, sex life status, and chronic diseases, ect, were collected confidentially. Participants will be primarily grouped according to the questionnaire interview at the enrollment visit by the investigators. Included in the questionnaires are relevant questions regarding inclusive criteria for three types of UI, which were designed according to the UI classification of the International Continence Society [1]. Participants will be included in the SUI group if question 5 is satisfied, in the UUI group if question 6 is satisfied, or in MUI if question 5 and 6 are both satisfied. All the communication between providers and participants during the questionnaire interview will be monitored by the PI and will be fully recorded for quality control analysis.

For grouping the participants, we will grade the disease situation for each of the participants grouped in each of the groups according to the symptoms using the Ingelman-Sundberg Indexing method and International Continence Society standards [1]. Grading criteria are listed in the following form (Table 1): Participants will be excluded if they are graded to have severe UI.

After collecting the above-mentioned data, all participants will completed a gynecological examination, one-hour urinal pad test, pelvic floor electrophysiological examination, uroflowmetry, pelvic floor ultrasound, and morphological examination (Table 2). All the examinations will be conducted by the teams, using the same model instruments to avoid data biases, and data will be recorded on tablets connected to the database. The items to be inspected are as follows:

(1) **Gynecological examination** includes uterus, vagina and appendages inspections; pressure inductive testing; bladder neck elevation testing; hand Oxford muscle strength classification testing; pelvic organ prolapse quantification (POP-Q); and one-hour urinal pad testing.
(2) **UI questionnaires** concerning International Consultation on Incontinence Questionnaire-Short Form (ICIQ-SF) for investigation of UI frequency, degree, and its impacts on quality of life via 0–21 scoring to represent the severity of the UI [22]; OABSS questionnaire for assessment of the symptoms of the participants whose bladders are overactive and the severities of their urinary frequency, nocturia, urinary urgency, and urinary incontinence via scoring 0–15 to indicate the lowest to highest severity [23,24]; and Urogenital Distress Inventory-6(UDI-6) for evaluation of the lower urinary tract dysfunction via scoring 0–24 to present the severity of the urinary incontinence [25,26].
(3) **Pelvic floor electrophysiological examination** on an instrument to collect data regarding indicators such as the maximum vaginal dynamic pressure, the vaginal resting pressure, the strength grades of type I and type II muscle, and the fatigue of type I and type II muscle fibers.
(4) **Uroflowmetry** on the maximum and average rate of urine flow, urination time, and the urine flow curve.
(5) **Pelvic floor ultrasound examination** performed by two attending doctors who have rich clinical experience of pelvic floor ultrasound for the residual urine, detrusor muscle thickness, bladder neck movement, urethral rotation angle, bladder posterior angle, the shape of the internal urethra opening, the distance from the lowest point of the bladder to the posterior lower edge of the pubic symphysis, levator ani muscle trauma, and levator ani hiatus area in the Valsalva status.
(6) **Morphological examination** a study reported that the pelvis architecture was closely related to UI in women, especially pelvic inlet and pelvic outlet diameters as risk factors for UI [27], therefore, we include pelvic floor morphology into the study, which mainly includes the inclination angle of the sacrum and the anterior superior iliac spine, and the pelvic tendency.

Enrollment and participant grouping will be completed after all of the above procedures. Participant follow-up will be conducted for 6 months, 12 months, and 18 months from enrollment by investigators who were trained for each follow-up question. The follow-ups will be conducted by telephone to learn UI progress by inquiring participants’ symptoms in terms of the history of urinary incontinence, the frequency of urine leakage, the urinary syndrome, the usage of urinal pad, and conscious impact on life, and using a manner of questionnaires (ICIQ-SF, OABSS, and UDI- 6) (Table 2). Patients whose situation could not be confirmed as progressive or who struggle to express their symptoms will be requested to return back to the hospital for follow-up. Follow-up will be terminated for participants whose UI symptoms have aggravated upgrading. Rehabilitation guidance will be provided to participants in the termination of follow-up. Follow-up will be continued for participants with no symptom aggravation for no more than three calls in a period of no longer than 18 months.

### 2.7. Study Endpoint

As there are no internationally accepted criteria for UI progress, we set up a series of criteria for referring literature, using the Ingelman-Sundberg Indexing method and International Continence Society [1] to judge UI progress in our study. The primary endpoint: UI progress will be judged if any of the following criteria are satisfied: For the SUI group, (1) urinary leakage inducive causes a change from coughing and sneezing to running, jumping and fast walking, or from running, jumping and fast walking to postural changes and rest at bed; (2) urinal pad usage changes from unneeded to needed; or (3) the baseline ICIQ-SF score is heightened for ≥4 [28]. For the UUI group, (1) OABSS score upgrades compared with the baseline, or (2) urinal pad usage changes from unneeded to needed. Progress will be confirmed for the MUI group if participants satisfy any of the progressive criteria for the SUI and UUI groups.

### 2.8. Data Management

All information from the study will be input into the computerized database developed by the Guangzhou Huibo Information Technology Co., Ltd. (Guangdong, China), under the study ID and together with the grouping data. Simultaneous double inputs by two independent providers will be adopted in order to match the two copies of the datasets so as to figure out any possible data error latterly. Data inputs will be managed by an experienced statistic professor.

### 2.9. Sample Size Consideration

According to one risk factor in the prediction model, 10 to 15 positive events (UI progress) are be required. It is preliminarily estimated that there are about 10 meaningful risk factors, so 100–150 cases need to be included in the study to get enough statistical power. In consideration of this, 15–20% of the patients were lost in follow-ups [6,9], and the number of participants in the training and validation groups are at a ratio of 2:1, at least 188–282 participants in total are required in order to have statistical power. As more than the anticipated number of targeted women expressed their willing for participation, we planned to recruit 800 participants at most in order to get greater power for the study.

### 2.10. Statistical Analysis and Progress Prediction Model Construction

Data analysis will be done using SAS^®^ 9.3 software (software installation point authorization number: 11202165).

We set up a prediction model based on the Cox regression model. According to univariate Cox regression analysis, risk factors with a *p* value < 0.05 will be selected to enter the model predictive factors, and then the forward method will be used to carry out multi-factor Cox regression analysis to build the prediction model of the UI disease progress, predicting the risk of UI disease progress. The subjects will be randomly divided into training and validation cohorts according to the ratio of 2:1. The discriminative ability of the prediction model will be analyzed through the area under the ROC curve, and the accuracy of the model will be evaluated by the Hosmer–Lemeshow decile chi-square test. The calibration of the ten models will be assessed with the predicted versus observed probability plots, and the discrimination of the models will be assessed with the prognostic separation D-statistic (D-statistic). The bootstrap method will be used to further internally verify the discriminative ability and accuracy of the model. External validation uses different hospitals for spatial validation to evaluate the extrapolation of the model. Moreover, 100 bootstrap random samplings will be performed to generate 100 bootstrap test sets. For each test set, the area under the ROC curve method and Hosmer–Lemeshow deciles will be used in the square test to objectively evaluate the stability and variation range of the model’s discriminative ability and accuracy. 

## 3. Discussion

UI is one of the chronic diseases that seriously affects middle-aged and elderly women in their quality of life. More and more women are suffering from UI. However, due to a lack of knowledge or willingness to seek medical treatment, many women, especially the elderly, develop moderate to severe UI before they visit a doctor for treatment. It has been recognized by the medical society that early detection is of great significance for the treatment of UI. Although a lot of risk factors are reported to be associated with UI, only some of these factors have been identified to reduce UI incidence and prevalence through their mitigation, which includes constipation, parity, gynecological diseases, or cardiovascular diseases [4,8]. UI prevalence and the likely associated factors have been widely reported by researchers from many countries. However, few studies have really focused on UI progress criteria and the factors that can potentially reverse UI, even slightly.

There are several studies on UI curation nowadays. A study by Zhishun Liu [19], a randomized controlled trial (RCT), showed that a greater than 50% decrease in terms of urinary leakage amount in SUI patients was achieved by treatment with electroacupuncture involving the lumbosacral region. A similar study by Renly Lim [28] on the curation of SUI reported a four-point ICIQ-SF score reduction in women with nonsurgical treatments for UI, which showed the minimum clinically important difference (MCID) with the baselines. A clinical study conducted by Liliana Giraldo-Rodríguez [9] on the epidemiology, progress, and predictive factors of UI in community-dwelling Mexican adults aged ≥ 50 indicated that depression and falls could decrease the incidence of UI, but they did not come up with criteria for UI curation or progress because of some limitations. In referring to the above studies, based on criteria for SUI disease curation, we thought backwards about one criterion of disease progress. We define “≥4 points of ICIQ-SF score increase” as one of the progress criteria for SUI progress, as it is more objective and convenient than the calculation of urine leakage or the frequency of urine leakage. The other criteria of SUI progress mainly adopt the Ingelman-Sundberg Indexing method. The criteria of UUI progress are defined by our team in consideration of the questionnaire of OABSS’s severity study on the UUI.

To date, there has been no published powerful clinical research on criteria for UI progress, suggesting that there is evidenced agreement on the risk-factors that can predict UI progress among the medical society. Meanwhile, there has been no research on disease progress models up to now, and the research on UI progress model is more meaningful for quality of life. Our work in this collaborative study aims to investigate the indicators most valuable for the prediction of UI development and progress through comparing the baseline and follow-up data for the same indicators, and, based on those data, by single factor and multi-factor Cox regression model analysis, we will try to develop a primary UI progress predication model for the world, which we believe will significantly contribute to the worldwide studies in this research area.

As screening for high-risk factors is mainly based on previous literature, many disease incidence prediction models are mainly based on questionnaires and basic gynecological examinations. The advantages of our study are that it includes morphology and ultrasound, electrophysiological examination, and other indicators that have rarely been performed. Those newly included indicators may provide early warning for UI progress and evidence for the early intervention and treatment of UI.

The UI predictive model will be developed to be an app compatible with mobile devices. When patients with symptoms of urine leakage enter relevant information and data via this app, the model system will automatically analyze their disease conditions to predict the potentiality of UI progress. It will serve to warm both the patients and the clinicians, remind the patients to seek medical treatments, and provide the clinicians evidence for preventive interventions. We believe that it could play an important role in alleviating the symptoms of urine leakage and promoting the recovery of UI.

The purpose for us recruiting patients from Beijing, a city in northern China, and Wuhan, a city in mid-southern China, is to ensure that the indicators and criteria could be determined from the analysis of the data from patients representing the northern and southern areas. If we succeed in the primary development of a predictive model for UI progress, the primary model should be applied in a large-scale study to demonstrate its effectiveness or to provide evidence for further modification.

Our study has some limits. Firstly, UI progress has not been clearly defined worldwide. The criteria for UI progress are defined by our team in referring a large amount of literature and the international recognized diagnostic criteria for UI and the questionnaire of ICIQ-SF and OABSS ’s study on UI. These criteria have been demonstrated to have certain clinical significance, but the relevant criteria for each of the indicators still need to be further discussed and explored in a large clinical sample research. Secondly, we design a convenient follow-up procedure via phone calls to reduce the drop-off rate. To avoid any possible information bias via phone calls, we trained the investigators before having them conduct the follow-ups, and will request patients who could not be diagnosed with progress to return to the hospital for follow-up confirmation. Thirdly, the progress years of UI are not clearly defined in the literature. Our study will project following the participants for up to 18 months, which is fine to observe UI progress, but is still not long enough to give powerful answers. We hope we can contribute towards a large-scale study in UI progress prediction through long-term follow-ups.

## 4. Conclusions

To the best of our knowledge, this is the first study to investigate the progress prediction model of associated risk factors for UI disease progress in elderly women. This study may provide more prediction information for elderly female UI patients and improve their quality of life. Information from this study could also be used to guide medical attention and to properly shape interventions for UI for older adults. The information regarding the problem of early diagnosis, treatment, and improvement of long-term prognosis among UI women is particularly useful, as there are few such studies in this UI progress area.

## Figures and Tables

**Table 1:** Diagnosis criteria for subtypes of UI.

UI Types	Grade	Criteria
SUI grading	Mild	UI appears when coughing or sneezing without the need for a urinal pad
	Moderate	UI appears when running, jumping, or fast walking, and a urinal pad is needed
	Severe	UI appears when changing body position or resting at bed
UUI grading		According to OABSS indicators Question 3 (urgency) has a score of 2 or more, and the total score is 3 or more)
	Mild	3 ≤ score ≤ 5
	Moderate	6 ≤ score ≤ 11
	Severe	Score ≥ 12
MUI grading		According to the grade of SUI and UUI related symptoms, highest grading to be matched

UI: urinary incontinence; SUI: stress urinary incontinence; UUI: urgency urinary incontinence; MUI: mixed urinary incontinence; OABSS: overactive bladder symptom score.

**Table 2:** Participant baseline screening, assessment, and follow-up schedule.

Characteristics	Baseline			Follow-Up (6 Months, 12 Months, 18 Months)		
	SUI	UUI	MUI	SUI	UUI	MUI
Age (≥60)	•	•	•			
Race	•	•	•			
Marital status	•	•	•			
Educational level	•	•	•			
Mainly physical labor	•	•	•			
BMI	•	•	•			
Parity	•	•	•			
Manner of delivery	•	•	•			
UI during pregnancy	•	•	•			
UI after childbirth	•	•	•			
Menopause	•	•	•			
Sex life	•	•	•			
Drink preference	•	•	•			
24-h Volume of liquid intake (mL)	•	•	•			
Smoking	•	•	•			
Comorbidities	•	•	•			
Chronic cough						
Asthma						
Diabetes						
Constipation						
Pelvic inflammatory disease	•	•	•			
Depression	•	•	•			
Urinary tract infection (last 4 weeks)	•	•	•			
History of gynecological surgery	•	•	•			
Family history of UI	•	•	•			
UI duration (year)	•	•	•	•	•	•
Frequency of urine leakage (time/month)	•	•	•	•	•	•
Symptoms accompaning urine leakage	•	•	•	•	•	•
Using urine pads (per/month)	▲	▲	▲	▲	▲	▲
Impact on life	•	•	•	•	•	•
POP-Q	•	•	•			
Hand test Oxford muscle strength grading	•	•	•			
One-hour urine pad test (g)	•	•	•			
Severity of UI	•	•	•	•	•	•
Mild						
Moderate						
Severe						
Pelvic floor electrophysiology examination	•	•	•			
Uroflowmetry	•	•	•			
Pelvic floor ultrasound	•	•	•			
Morphological examination	•	•	•			
ICIQ-SF score	•	•	•	•	•	•
OABSS score	•	•	•	•	•	•
UDI-6 score	•	•	•	•	•	•
UI progress				▲	▲	▲

•: Indicates mandatory items; ▲: Investigator will decide whether to perform the test according to clinical signs or clinical evaluation. UI: urinary incontinence; SUI: stress urinary incontinence; UUI: urgency urinary incontinence; MUI: mixed urinary incontinence; OABSS: overactive bladder symptom score; POP-Q: pelvic organ prolapse quantification; ICIQ-SF: International Consultation on Incontinence Questionnaire-short form; UDI-6: Urogenital Distress Inventory-6; BMI: body mass index.

## Data Availability

Not applicable.

## Abbreviations

UI: urinary incontinence; SUI: stress urinary incontinence; UUI: urgency urinary incontinence; MUI: mixed urinary incontinence; POP-Q: pelvic organ prolapse quantification; ICIQ-SF: International Consultation on Incontinence Questionnaire-short form; OABSS: overactive bladder symptom score; UDI-6: Urogenital Distress Inventory-6; BMI: body mass index; RCT: Randomized Controlled Trial; MCID: minimum clinically important difference.
